# Impact of cryopreservation on immune cell subset frequencies in human peripheral blood mononuclear cells following assessment of monoclonal antibodies to CD39 and P2X7

**DOI:** 10.1007/s11302-026-10180-4

**Published:** 2026-09-07

**Authors:** Leon Koning, Ashleigh Splitt, Daniel Brungs, Martin G. Carolan, Senthilkumar Gandhidasan, Ronald Sluyter

**Affiliations:** 1 Molecular Horizons, University of Wollongong, Wollongong, NSW 2522 Australia; 2 School of Science, Faculty of Science, Medicine and Health, University of Wollongong, Wollongong, NSW 2522 Australia; 3 Illawarra Cancer Care Centre, Wollongong Hospital, Wollongong, NSW 2500 Australia; 4 Graduate School of Medicine, Faculty of Science, Medicine and Health, University of Wollongong, Wollongong, NSW 2500 Australia

**Keywords:** Purinergic signalling, Cryopreservation, CD39, *ENTPD1*, P2X7, *P2RX7*

## Abstract

Peripheral blood mononuclear cells (PBMCs) are commonly cryopreserved for immune cell profiling analyses, like flow cytometry. Cryopreservation of PBMCs may affect the recovery of immune cell subsets or the presence of cell-surface molecules, potentially skewing results compared to freshly isolated PBMCs. The ectonucleotidase CD39 promotes immunosuppression through the hydrolysis of extracellular nucleotides such as adenosine 5’-triphosphate (ATP) to limit P2X7 activation and downstream immune responses. Because of this, CD39 and P2X7 are emerging as potential biomarkers in cancer and other diseases. Using flow cytometry, this study demonstrated that an anti-human CD39 monoclonal antibody (mAb) (clone TU66) bound human embryonic kidney (HEK) 293 cells transfected with human CD39, but not mock-transfected HEK293 cells. In contrast, an anti-human P2X7 mAb (clone 1058613) bound similarly to HEK293 cells transfected with human P2X7 and non-transfected HEK293 cells. Using flow cytometry, this study then assessed the impact of cryopreservation on the population frequencies of immune cell subsets including CD39^+^ cells in human PBMC samples. These analyses revealed that cryopreservation had a minor, yet statistically significant impact on the population frequencies of CD4^+^, CD8^+^ and CD4^−^CD8^−^ T cells. Cryopreservation also had a significant impact on the frequencies of CD39^+^CD19^+^ B cells and CD39^+^ non-classical monocytes. Overall, this study informs researchers and biobank workers about the need to consider the impact of cryopreservation when interpreting data from stored samples.

**Supplementary Information:**

The online version contains supplementary material available at https://doi.org/10.1007/s11302-026-10180-4.

## Introduction

Cryopreservation is widely used to preserve immune cells, such as peripheral blood mononuclear cells (PBMCs), for subsequent use in flow cytometry or other analyses. Cell samples are cooled to extreme temperatures to preserve their cell state by slowing down their intrinsic biological and chemical processes. However, cryopreservation has limitations, particularly during the transition phase from liquid to frozen states. During this process, freezing causes the formation of ice crystals that can damage cellular structures, which may impact cell viability and function [1]. Various measures are taken by researchers and biobank workers to limit damage to cells, such as controlling the freezing and thawing rates and using cryoprotective agents. Cryoprotectants, such as dimethyl sulfoxide (DMSO) or glycerol are added to the cells before freezing to limit the formation of the damaging ice crystals [2]. Even though these measures limit the damage to the cells, studies have shown that cryopreservation may affect the population frequencies of immune cell populations and the expression of immune cell markers [3]. As such purinergic signalling cell-surface molecules may also be affected by cryopreservation, but this remains largely unexplored.

The purinergic signalling pathway was proposed by the late Geoffrey Burnstock [4]. In this pathway, extracellular nucleotides, including adenosine 5’-triphosphate (ATP) or adenosine 5’-diphosphate (ADP) activate purinergic receptors to regulate immune and other physiological responses [5]. Further, ectonucleotidases can degrade these nucleotides to reduce purinergic receptor activation [6]. For example, CD39 is an ectonucleotidase that hydrolyses ATP and ADP to adenosine 5’-monophosphate (AMP), causing immunosuppressive effects. This reduction in extracellular concentrations of ATP and ADP limits the activation of pro-inflammatory P2 receptors, most notably P2X7 in various cell types [7–10] and disease models [11, 12]. Moreover, the resulting AMP is hydrolysed by CD73 to adenosine, which activates anti-inflammatory P1 receptors [13].

Due to the immunoregulatory role of CD39, this ectonucleotidase has been implicated in a wide range of diseases and disorders, including sepsis, autoimmune and neurodegenerative disorders, and cancer [14]. Therefore, CD39 is emerging as a potential biomarker in these and other diseases [15]. While studies have shown that CD39 is present on immune cells, including PBMCs [16–19], there are limited studies reporting the presence of CD39 on T and B cell, natural killer (NK) cell, and monocyte subsets. Moreover, there are limited studies examining the impact of cryopreservation on the frequency of CD39^+^ cells in PBMCs. Amongst these, most focused on the major T and B cell populations [20–23] with no studies assessing the effects of cryopreservation on the frequency of CD39^+^ cells in CD4^+^CD8^+^ or CD4^−^CD8^−^ T cell, NK cell or monocyte subsets.

P2X7 is also emerging as a potential biomarker [24], but its exploration has been limited by the lack of a commercially purified monoclonal antibody (mAb). Recently, an anti-human P2X7 mAb (clone 1058613) in purified or fluorochrome-conjugated formats has become available (R&D Systems, Minneapolis, USA). However, to the best of our knowledge, this mAb is yet to be reported in any publications.

Using flow cytometry, this study demonstrated that an anti-human CD39 mAb (clone TU66) bound human embryonic kidney (HEK) 293 cells transfected with human CD39, but not mock-transfected HEK293 cells. In contrast, an anti-human P2X7 mAb (clone 1058613) bound similarly to HEK293 cells transfected with human P2X7 and non-transfected HEK293 cells. Moreover, this study assessed the impact of cryopreservation on the population frequencies and CD39^+^ immune cell subsets in human PBMC samples, demonstrating mostly minor, yet statistically significant changes in some population frequencies including CD39^+^ subsets.

## Materials and methods

### Materials

Dulbecco’s Modified Eagle Medium/Nutrient Mixture F12 (DMEM/F12), GlutaMAX, penicillin, streptomycin, geneticin, Dulbecco’s phosphate-buffered saline (PBS), 0.05% trypsin-ethylenediaminetetraacetic acid (EDTA), Opti-MEM, Lipofectamine 3000, Hoechst 33342 nucleic acid stain and Roswell Park Memorial Institute (RPMI)-1640 medium were from Thermo Fisher Scientific (Waltham, USA). Foetal bovine serum (FBS) (heat-inactivated before use) was from CellSera Australia (Rutherford, Australia). PcDNA3.1(+) plasmid encoding for human CD39 (UniProt accession number P49961-1) (human CD39 plasmid) was obtained from Gene Universal Inc (Newark, USA). Lymphoprep was from STEMCELL Technologies (Vancouver, Canada). Trypan blue solution, DMSO and isopropanol were from Sigma-Aldrich (St. Louis, USA). Human FcR Blocking Reagent was from Miltenyi Biotec (Bergisch Gladbach, Germany). Fluorochrome-conjugated mAbs are listed in Table 1.

**Table 1:** Fluorochrome-conjugated mAb information

Target	Clone	Fluorochrome^a^	Excitation Laser/Emission Filter (nm)^b^	Manufacturer^c^
CD3	UCHT1	BV711	405/710_50	BD Bioscience
CD4	SK3	BV605	405/610_20	BD Bioscience
CD8	RPA-T8	BV421	405/450_50	BD Bioscience
CD16	3G8	PE	561/586_15	BD Bioscience
CD56	B159	PE-Cy7	561/780_60	BD Bioscience
CD19	HIB19	PerCP-Cy5.5	488/695_40	BD Bioscience
CD14	M5E2	FITC	488/525_50	BD Bioscience
CD39	TU66	APC	640/670_30	BD Bioscience
P2X7	1058613	Alexa Fluor 647	640/670_30	R&D Systems
P2X7 isotype control	MOPC-173	Alexa Fluor 647	640/670_30	BD Bioscience
P2X7	L4	DyLight 488	488/525_50	In-house^d^

^a^Abbreviations: *APC* allophycocyanin, *BV* Brilliant Violet, *Cy* cyanine, *FITC* fluorescein isothiocyanate, *PE* phycoerythrin, *PerCP* peridinin-chlorophyll-protein complex

^b^Hoechst 33342 nucleic acid stain and Zombie NIR live-dead stain were collected at 405/450_50 and 640/780_60 nm, respectively

^c^BD Bioscience (San Diego, USA); R&D Systems (Minneapolis, USA)

^d^Purified and conjugated to DyLight 488 as described [26]

### Cell lines

HEK293 cells were originally from the American Type Culture Collection (Manassas, USA) and maintained in DMEM/F12 containing 10% FBS, 2 mM GlutaMAX, 100 U/mL penicillin and 100 mg/mL streptomycin (complete medium). HEK293 cells stably transfected with human P2X7 (HEK-P2X7) [25] were originally provided by Leanne Stokes (University of East Anglia, Norwich, UK) and maintained in complete medium containing 400 µg/mL geneticin. All cells were cultured in 25 cm^2^ tissue culture flasks (Greiner Bio-One, Kremsmünster, Austria) at 37 °C with 5% CO_2_ using an MCO-170AC-PE IncuSafe CO_2_ Incubator (PHC Holdings Corporation, Tokyo, Japan). All subsequent incubations at 37 °C with 5% CO_2_ were performed with this instrument. When cells achieved 90–95% confluency, they were washed twice with 1 mL of PBS. The cells were then released from the flask surface with 1 mL of 0.05% trypsin–EDTA at 37 °C with 5% CO_2_ for 3 min. Trypsin was neutralised with 5 mL corresponding complete medium and the cells centrifuged at 150 × *g* for 5 min. Harvested cells were then subcultured or used in experiments. Cells were routinely tested and confirmed to be negative for *Mycoplasma* spp. contamination using the MycoAlert Mycoplasma Detection Kit (Lonza, Basel, Switzerland).

### Preparation of the human CD39 plasmid

The human CD39 plasmid was transformed into DH5α *Escherichia coli* (Thermo Fisher Scientific) using standard techniques and isolated with the Wizard Plus SV Minipreps DNA Purification System (Promega, Madison, USA) according to the manufacturer's instructions. Plasmid concentration was determined using a NanoDrop One/OneC Microvolume UV–Vis Spectrophotometer (Thermo Fisher Scientific).

### Transfection of HEK293 with human CD39

Harvested HEK293 cells were plated at a concentration of 2.5 × 10^5^ cells/well in complete medium (2 mL/well) in 6-well plates (Corning, Corning, USA) and incubated at 37 °C with 5% CO_2_ for 24 h until 75% confluent. For each required well, 250 µL of Opti-MEM with 5 μL Lipofectamine 3000 Reagent and 4 µL P3000 Reagent and either 0.4 µg of the human CD39 plasmid or no plasmid (mock transfection) was prepared in 1.5 mL microfuge tubes (Sarstedt, Nümbrecht, Germany) and incubated for 15 min at room temperature (RT). Following incubation, mixtures were added to their respective wells in a drop-wise fashion and the plate manually agitated for 5 s. The cells were then incubated at 37 °C with 5% CO_2_ for 24 h. After incubation, the cells were washed with 1 mL of PBS and incubated with 1 mL of 0.05% trypsin–EDTA at 37 °C with 5% CO_2_ for 3 min. The trypsin was then neutralised with 2 mL of complete medium and the cells centrifuged at 150 × g for 5 min. Cells were then used for flow cytometric analyses.

### Human blood collection

All human blood collections and experiments were conducted with informed, written consent from donors and in accordance with the Human Research Ethics Committee at the University of Wollongong (Wollongong, Australia). Peripheral blood (~ 20 mL per donor) was collected from five healthy donors (four males, one female; African or Western European descent; aged 23–54 years; mean age 35 years) into K3E K3EDTA VACUETTE tubes (Greiner Bio-One). Blood samples were used for the isolation of PBMCs by SepMate density gradient separation within 2 h of collection.

### Isolation of PBMCs from human blood

Lymphoprep at RT was pipetted through the insert hole of the SepMate tubes (STEMCELL Technologies) before being slowly layered with peripheral blood diluted in equal volumes of RT PBS. SepMate tubes were then centrifuged on a Heraeus Multifuge X3R (Thermo Fisher Scientific) at 1200 × *g* for 10 min at 21 °C with acceleration set to 9 and deceleration at 5. Subsequent centrifugations were performed with the same instrument, but with acceleration and deceleration set to 9. Supernatants, consisting of PBMCs and PBS-diluted blood plasma, were transferred into 50 mL conical-bottom centrifuge tubes (Greiner Bio-One) and filled with RPMI-1640 medium containing 10% FBS at 4 °C. Tubes were then centrifuged at 400 × *g* for 10 min at 4 °C. PBMCs were then resuspended in 10 mL RPMI-1640 medium containing 10% FBS at 4 °C. PBMCs were enumerated and assessed for viability using a haemocytometer (Marienfeld Superior, Lauda-Königshofen, Germany) following dilution of 100 µL PBMCs in 100 µL trypan blue solution. PBMCs were then cryopreserved or used for flow cytometric analyses as follows.

### Cryopreservation of human PBMCs

Isolated PBMCs, in 10 mL RPMI-1640 medium containing 10% FBS at 4 °C, were centrifuged at 300 × *g* for 10 min at 4 °C. PBMCs were resuspended in RPMI-1640 medium containing 10% FBS at a concentration of 4–10 × 10^6^ cells/mL. An equal volume of ice-cold freeze down medium, consisting of 40% RPMI-1640 medium, 40% FBS and 20% DMSO, was slowly added to the cell suspension with swirling. PBMCs (2–5 × 10^6^ cells) were then aliquoted in volumes of 1 mL into 1.8 mL CryoPure tubes (Sarstedt). PBMCs were frozen in a Nalgene Cryo 1 °C freezing container (Nalgene, Rochester, USA) containing isopropanol in a −80 °C freezer (Snijders Labs, Tilburg, The Netherlands) for 1–2 days. PBMCs were then stored in the vapour phase in a liquid nitrogen storage tank (Chart Industries, Ball Ground, USA) until required.

### Immunolabelling and flow cytometric analyses of cell lines

To prepare HEK293 and transfected HEK-293 cells for flow cytometric analyses, harvested cells were washed twice with 10 mL PBS containing 2% FBS at 150 × *g* for 5 min at 4 °C. Cells, enumerated using a haemocytometer (as above), were resuspended in PBS containing 2% FBS at 1 × 10^7^ cells/mL. Cells (100 µL/tube) were aliquoted into 5 mL round-bottom tubes (Sarstedt) and incubated with 4 µL of human FcR Blocking Reagent for 10 min on ice. Cells were then incubated in the absence (fluorescent minus one (FMO)) or presence of fluorochrome-conjugated mAbs (as indicated) and 1 µg Hoechst 33342 nucleic acid stain for 15 min on ice protected from light. Cells were then washed with 1 mL PBS at 300 × *g* for 3 min at 4 °C and resuspended in 250 µL PBS. Events were acquired by a LSR Fortessa X-20 flow cytometer (BD Biosciences, San Jose, USA) with corresponding excitation lasers and emission filters (Table 1) using FACSDiva software version 8.0 (BD Biosciences). Data were analysed by FlowJo software version 10.10.0 (BD Biosciences). The percentage of CD39^+^ or P2X7^+^ cells was determined by setting the marker to ~ 2% positive cells for each FMO group and applying that marker to the respective sample groups [27].

### Immunolabelling and flow cytometric analyses of human PBMCs

After cryopreservation for approximately 1 or 6 months, PBMC samples were thawed in a 37 °C water bath. Thawed PBMCs were transferred to 15 mL conical-bottom centrifuge tubes (Greiner Bio-One) with 9 mL of RPMI-1640 medium containing 10% FBS, and centrifuged at 300 × *g* for 5 min at 4 °C. Fresh or thawed PBMCs were washed in 10 mL of PBS at 300 × *g* for 5 min at 4 °C. PBMCs in PBS (100 µL) were aliquoted equally into 5 mL round-bottom tubes and incubated with 1 µL of Zombie NIR live-dead stain from the Zombie NIR Viability Kit (BioLegend, San Diego, USA) for 15 min on ice protected from light. PBMCs were then washed with 2 mL of PBS containing 2% FBS at 300 × *g* for 3 min at 4 °C. Then, 20 µL of human FcR Blocking Reagent was added to each tube and incubated for 10 min on ice protected from light. PBMCs were then incubated in the absence (FMO) or presence of fluorochrome-conjugated mAbs (at the manufacturers’ test volumes) for 15 min on ice protected from light. PBMCs were washed twice with 1 mL PBS at 300 × *g* for 3 min at 4 °C and resuspended in 250 µL PBS. Events were acquired by a LSR Fortessa X-20 flow cytometer with corresponding excitation lasers and emission filters (Table 1) using FACSDiva software version 8.0. Data were analysed by FlowJo software version 10.10.0. The percentage of immune cells subsets was determined from the shown gating strategies and presented as a percentage of parent populations as indicated. The percentage of CD39^+^ cells was determined by setting the marker to ~ 2% positive cells for each FMO group and applying that marker to the respective sample groups.

### Statistical analysis

All statistics were calculated using Prism version 10.6.0 (GraphPad Software, Boston, USA). Data were tested for normality using a Shapiro–Wilk test. Normally distributed data were analysed using a repeated-measures one-way ANOVA with Geisser-Greenhouse correction, followed by Dunnett’s multiple comparisons test to compare 1- and 6-month cryopreserved samples with fresh samples. Non-normally distributed data were analysed using a Friedman test, followed by Dunn’s multiple comparisons test for the same comparisons. Changes after cryopreservation were regarded as significant if *p* < 0.05.

## Results

### The anti-CD39 mAb (clone TU66) binds to human CD39- but not mock-transfected HEK293 cells

**Fig. 1:**
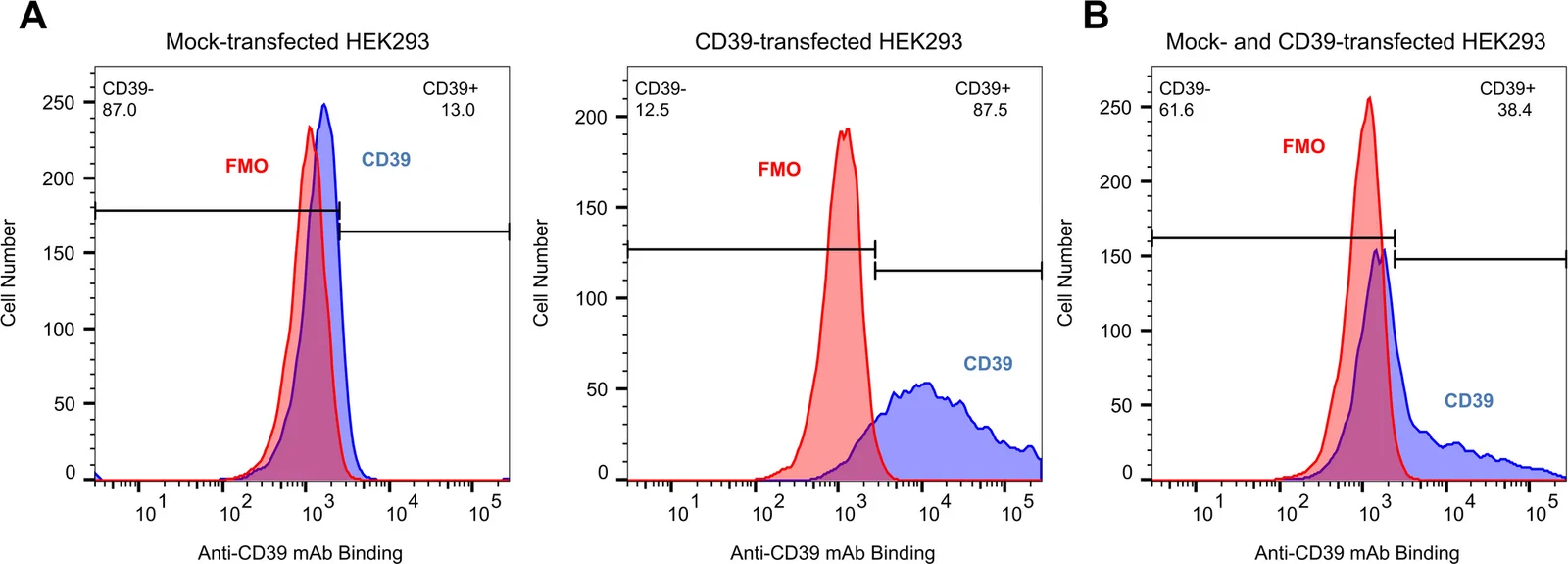
The anti-human CD39 mAb (clone TU66) binds to human CD39- but not mock-transfected HEK293 cells. (**A**, **B**) HEK293 cells were transfected with human CD39 plasmid DNA or mock transfected. At 24 h post-transfection (**A**) each cell type or (**B**) both cell types mixed 1:1 were incubated in the absence (FMO) or presence of the APC-conjugated anti-human CD39 mAb (clone TU66) and Hoechst 33342 nucleic acid stain and analysed by flow cytometry. The marker was set to ~ 2% positive cells for each FMO group, with the difference between mAb binding and the corresponding FMO used to determine the percentage of CD39^+^ cells. (**A**, **B**) Results are from one experiment

The anti-human CD39 mAb (clone TU66) has been extensively used to assess CD39 on immune cell subsets [28–30], but to the best of our knowledge, the binding of this mAb to CD39 has not been validated independently of the companies which produce this mAb. To validate the use of the anti-human CD39 mAb (clone TU66) in subsequent flow cytometric immune cell analyses, this mAb was tested against mock- and human CD39-transfected HEK293 cells. HEK293 cells are used in CD39 expression studies due to minimal endogenous expression of this ectonucleotidase [31] and ease of transfection [32]. Each cell type, or both cell types mixed at a 1:1 ratio, were incubated in absence (FMO) or presence of the allophycocyanin (APC)-conjugated anti-CD39 mAb (clone TU66). Cells were analysed by flow cytometry and gated using a consistent gating strategy as per Figure S1. Mock-transfected HEK293 cells showed minimal binding of anti-CD39 mAb binding compared to the FMO group (Fig. 1A). In contrast, CD39-transfected HEK293 cells showed a large shift to the right in fluorescence for the anti-CD39 mAb-stained group compared to the FMO group. This indicated that the anti-CD39 mAb bound CD39-transfected cells, but not mock-transfected cells. When mock- and CD39-transfected HEK293 cells were mixed 1:1, the CD39-stained group displayed two distinct populations: one population with minimal binding similar to the FMO group above (Fig. 1A), and a second population with higher anti-CD39 mAb binding (Fig. 1B). This is consistent with the notion that the anti-CD39 mAb selectively binds CD39.

### The anti-P2X7 mAb (clone 1058613) binds similarly to non-transfected HEK293 and HEK-P2X7 cells

**Fig. 2:**
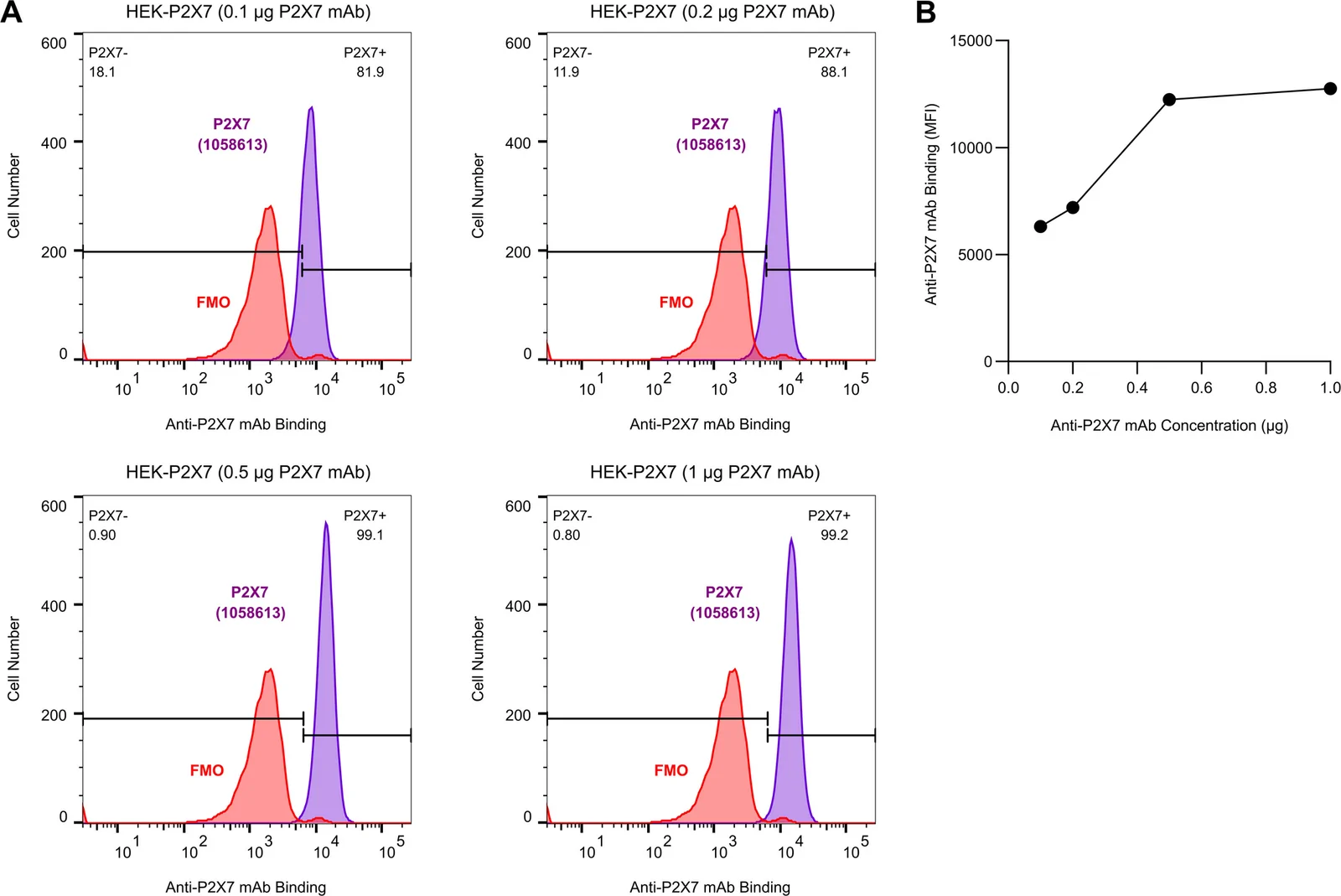
Titration of the anti-human P2X7 mAb (clone 1058613) shows maximal binding at 0.5 to 1 µg per test. (**A, B**) HEK-P2X7 cells were incubated in the absence (FMO) or presence of 0.1 µg, 0.2 µg, 0.5 µg, or 1 µg of the Alexa Fluor 647-conjugated anti-human P2X7 mAb (clone 1058613) and Hoechst 33342 nucleic acid stain and analysed by flow cytometry. (**A**) The marker was set to ~ 2% positive cells for each FMO group, with the difference between mAb binding and the corresponding FMO used to determine the percentage of P2X7^+^ cells. (**B**) The difference in MFI between the anti-P2X7 mAb and the FMO was used to determine anti-P2X7 mAb binding (**A**, **B**) Results are from one experiment

The anti-human P2X7 mAb (clone 1058613) has recently become commercially available but, to the best of our knowledge, use of this mAb has not been reported in the literature. To validate this mAb, HEK-P2X7 cells were incubated in absence (FMO) or presence of 0.1 µg, 0.2 µg, 0.5 µg, or 1 µg of the Alexa Fluor 647-conjugated anti-P2X7 mAb and analysed by flow cytometry. All mAb groups showed a large shift to the right compared to the FMO group, indicating binding of the anti-P2X7 mAb to the HEK-P2X7 cells (Fig. 2A). The median fluorescent intensity (MFI) of the FMO group was deducted from the MFI of the mAb-stained group to determine relative anti-P2X7 mAb binding. The anti-P2X7 mAb showed maximal binding at 0.5 µg to 1 µg (Fig. 2B). As the samples stained with 1 µg of anti-P2X7 mAb showed maximal binding, this concentration was used for subsequent experiments.

**Fig. 3:**
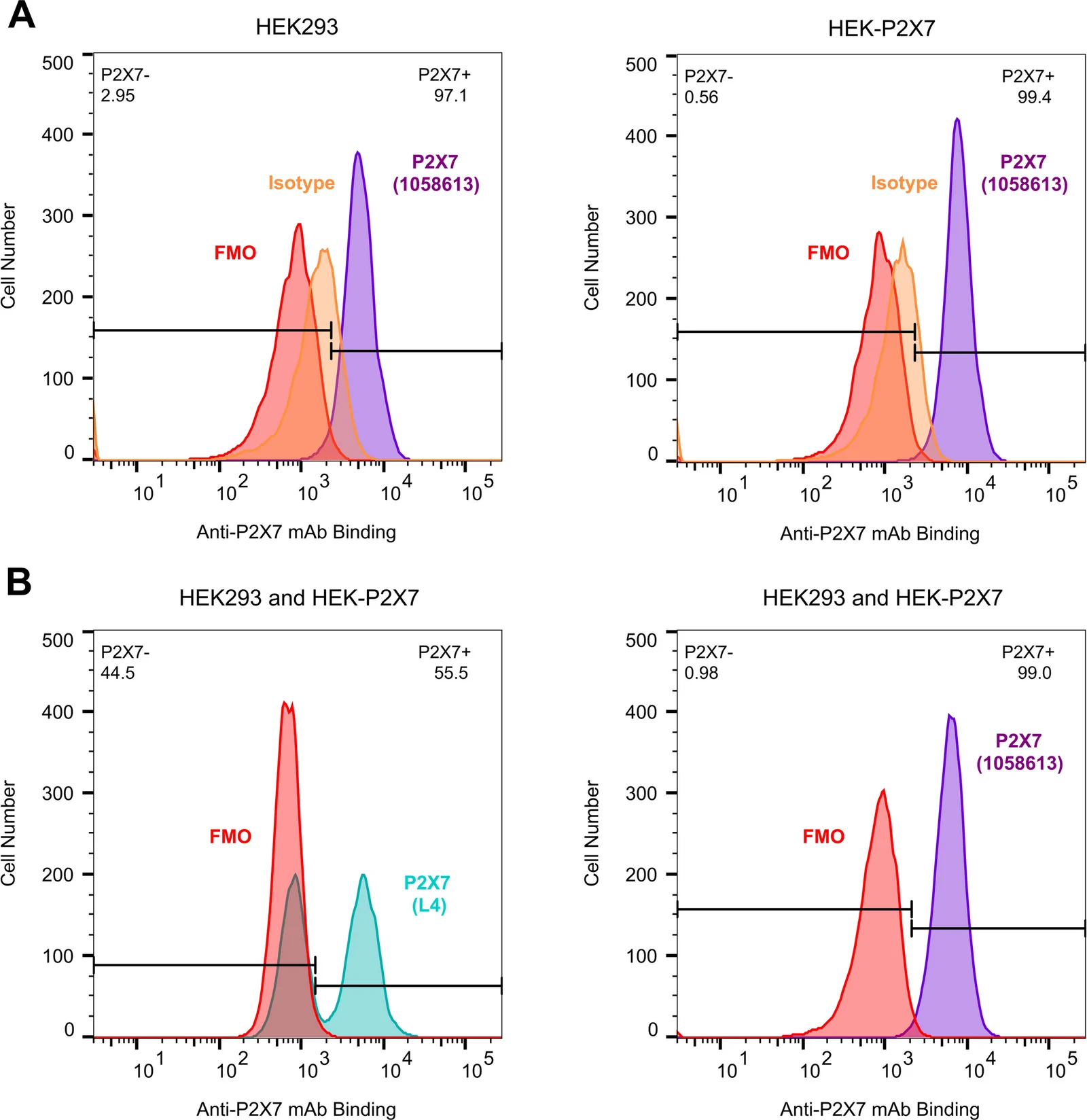
The anti-human P2X7 mAb (clone 1058613) binds similarly to HEK293 and HEK-P2X7 cells. (**A**) Non-transfected HEK293 or HEK-P2X7 cells were incubated in the absence (FMO) or presence of 1 µg of the Alexa Fluor 647-conjugated anti-human P2X7 mAb (clone 1058613) or isotype control mAb (clone MOPC-173) and analysed by flow cytometry. (**B**) HEK293 and HEK-P2X7 cells mixed 1:1 were incubated in the absence (FMO) or presence of 1 µg of the Alexa Fluor 647-conjugated anti-human P2X7 mAb (clone 1058613) or the DyLight 488-conjugated anti-human P2X7 mAb (clone L4) and Hoechst 33342 nucleic acid stain and analysed by flow cytometry. (**A**, **B**) The marker was set to ~ 2% positive cells for each FMO group, with the difference between anti-P2X7 mAb binding and the corresponding FMO used to determine the percentage of P2X7^+^ cells. Results are from one experiment

To further validate the anti-P2X7 mAb (clone 1058613), non-transfected HEK293 cells, which are P2X7 null [33], and HEK-P2X7 cells were incubated in the absence (FMO) or presence of the anti-human P2X7 mAb or isotype control mAb (clone MOPC-173) and analysed by flow cytometry (Fig. 3A). Unexpectedly, both cell types incubated with the anti-P2X7 mAb showed large and similar shifts to the right in fluorescence compared to the FMO group. For both cell types, the isotype control mAb groups showed minor but similar shifts to the right compared to the FMO group. This suggests that the anti-P2X7 mAb does not selectively bind to human P2X7.

To test the above observation (Fig. 2A), non-transfected HEK293 and HEK-P2X7 cells were mixed at a 1:1 ratio and incubated in the absence (FMO) or presence of either the fluorochrome-conjugated anti-P2X7 mAb (clone 1058613) or anti-P2X7 mAb (clone L4), which we [34] and others [35] have shown selectively binds to human P2X7, as a positive control. Samples were then analysed by flow cytometry. Mixed HEK293 and HEK-P2X7 cells incubated with the anti-P2X7 mAb (clone L4) displayed two distinct populations. One population with similar fluorescence to the FMO, and a second population with a large shift to the right in fluorescence compared to the FMO (Fig. 3B). In contrast, mixed HEK293 and HEK-P2X7 cells incubated with the anti-P2X7 mAb (clone 1058613) revealed a single population with a large shift to the right in fluorescence compared to the FMO. This suggested that the anti-P2X7 mAb (clone 1058613) binds non-specifically. To test that this was unrelated to the amount of mAb used, HEK293 and HEK-P2X7 cells, mixed 1:1, were incubated in absence (FMO) or presence of 0.02 µg to 2 µg of this mAb. As for 1 µg mAb above (Fig. 3B), incubation of cells with 0.02 µg to 2 µg mAb revealed a single population with an increasing shift to the right in fluorescence compared to the FMO (Fig. S2).

Collectively, this data indicates that the anti-P2X7 mAb (clone 1058613) does not selectively bind human P2X7 and is unable to discriminate P2X7^+^ cells from P2X7^−^ cells under the conditions used in this study. In the absence of a suitable commercially available anti-P2X7 mAb, P2X7 was omitted from the subsequent flow cytometric immune cell analyses.

### Cryopreservation of PBMCs for up to 6 months causes minimal, yet statistically significant changes in population frequencies of CD4^+^, CD8^+^ and CD4^−^CD8^−^ T cells

**Fig. 4:**
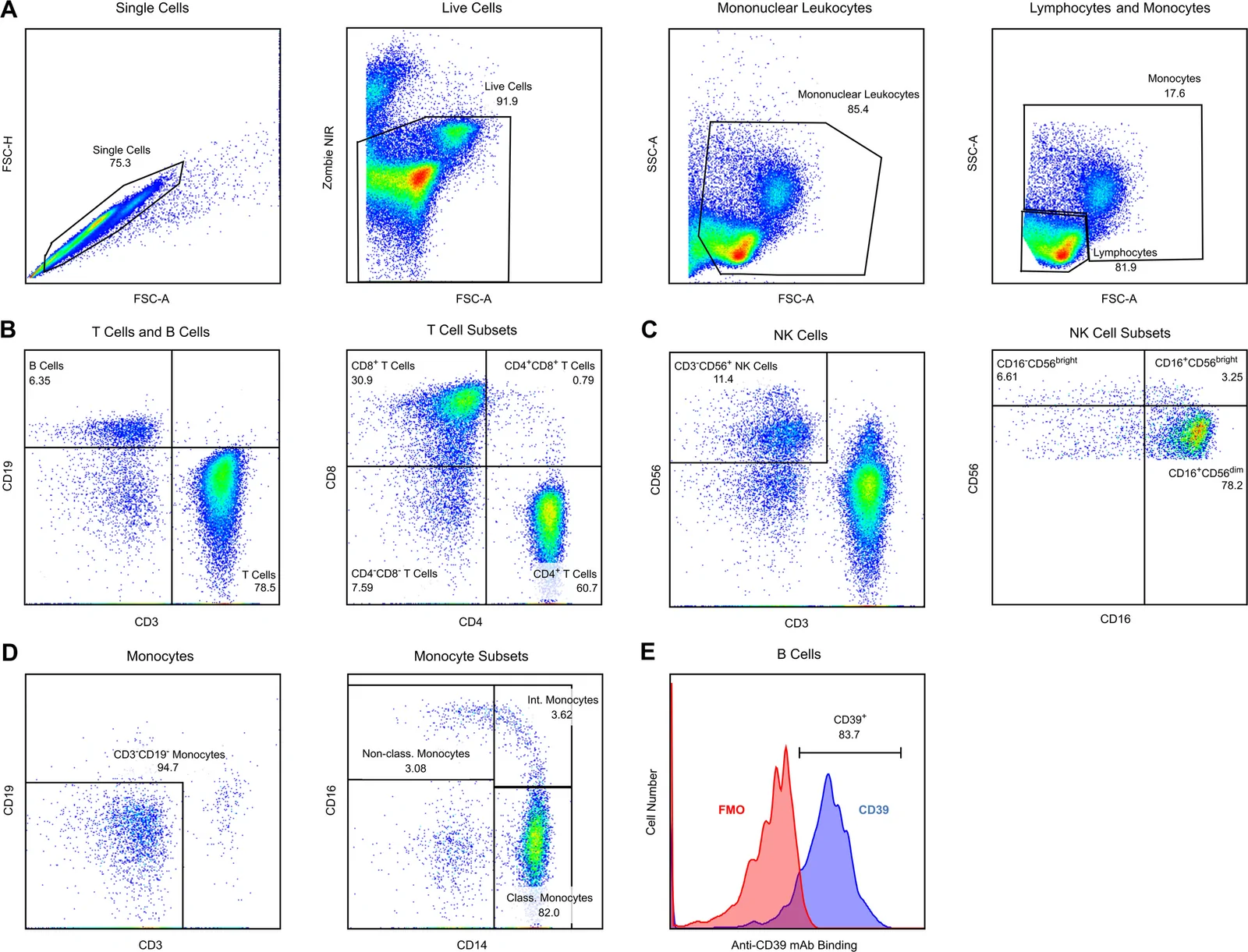
Representative gating strategy to identify immune cell subsets including CD39^+^ cells. (**A**) Fresh or cryopreserved PBMCs were stained with Zombie NIR live-dead stain and fluorochrome-conjugated mAbs and analysed by flow cytometry. Single cells were gated by forward-scatter area (FSC-A) and height (FSC-H). Live cells were gated by using the Zombie NIR live-dead stain. FSC-A and side-scatter area (SSC-A) were used to gate mononuclear leukocytes and then lymphocytes or monocytes. (**B**) CD3^+^CD19^−^ T cells and CD3^−^CD19^+^ B cells were gated from lymphocytes. CD4^+^CD8^−^, CD4^−^CD8^+^, CD4^+^CD8^+^ and CD4^−^CD8^−^ T cells were gated from CD3^+^CD19^−^ T cells. (**C**) CD16^+^CD56^dim^, CD16^+^CD56^bright^, and CD16^−^CD56^bright^ NK cells were gated from CD3^−^CD56^+^ NK cells. (**D**) CD14^+^CD16^−^ classical (class.) monocytes, CD14^+^CD16^+^ intermediate (int.) monocytes and CD14^−^CD16^+^ non-classical (non-class.) monocytes were gated from CD3^−^CD19^−^ monocytes. (**E**) The marker was set to ~ 2% positive cells for each FMO group, with the difference between anti-CD39 mAb binding and the corresponding FMO used to determine the percentage of CD39^+^ cells. An example for 6-month cryopreserved CD3^−^CD19^+^ B cells is shown

To determine the impact of cryopreservation on the population frequencies of immune cell subsets in human PBMC samples, PBMCs from five healthy donors were freshly isolated from whole blood using SepMate density gradient separation and analysed either immediately (fresh samples) or after 1- or 6-month cryopreservation. Fresh or cryopreserved PBMCs were stained with fluorochrome-conjugated mAbs and analysed by flow cytometry using a consistent gating strategy (Fig. 4A-D).

**Fig. 5:**
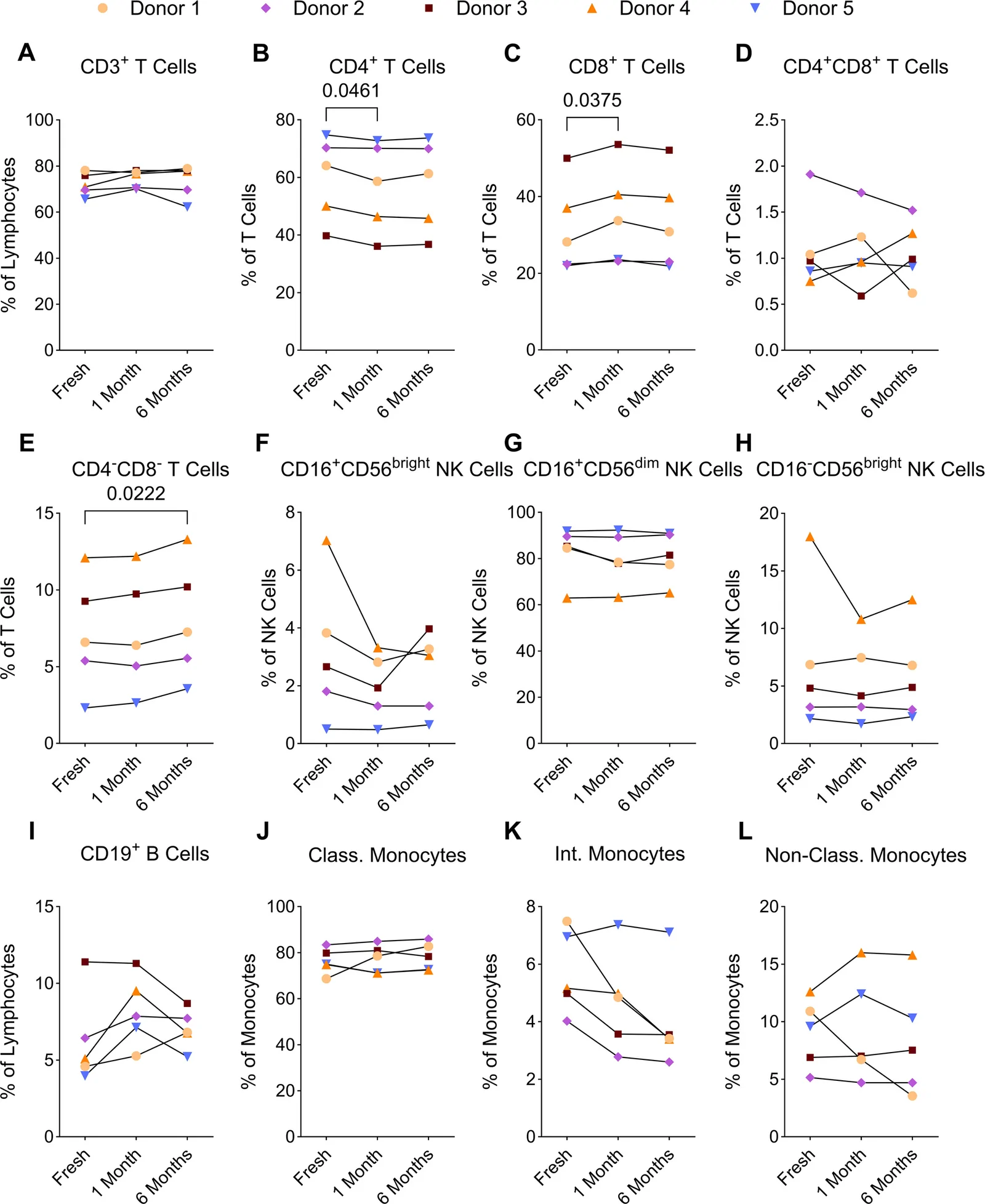
Cryopreservation of PBMCs for up to 6 months causes minimal, yet statistically significant changes in population frequencies of CD4^+^, CD8^+^ and CD4^−^CD8^−^ T cells. PBMCs from five healthy donors were freshly isolated from whole blood using SepMate density gradient separation and analysed either immediately (fresh) or after cryopreservation for 1 or 6 months. Fresh or cryopreserved PBMCs were stained with Zombie NIR live-dead stain and fluorochrome-conjugated mAbs and analysed by flow cytometry. Population frequencies are shown as percentages of (**A**, **I**) total lymphocytes, (**B**-**E**) total CD3^+^CD19^−^ T cells, (**F**–**H**) total CD3^−^CD56^+^ NK cells, and (**J**-**L**) total monocytes. Data were tested for normality using a Shapiro–Wilk test. Normally distributed data were analysed using a (**A**-**C**,** E**-**J**,** L**) repeated-measures one-way ANOVA with Geisser-Greenhouse correction and a Dunnett’s multiple comparisons test. Non-normally distributed data were analysed using a (**D**,** K**) Friedman test, followed by a Dunn’s multiple comparisons test

Population frequencies of T cell, NK cell and monocyte subsets and B cells following cryopreservation for 1 or 6 months were compared to fresh samples. For T cell subsets, no significant changes in population frequencies were observed between fresh and cryopreserved samples for total CD3^+^ T cells (Fig. 5A) and CD4^+^CD8^+^ T cells (Fig. 5D). In contrast, a minimal yet statistically significant decrease was observed at 1-month cryopreservation in the frequencies of CD4^+^ T cells (5.0% decrease, *p* = 0.0461) (Fig. 5B). Conversely, frequencies of CD8^+^ T cells (9.4% increase, *p* = 0.0375) (Fig. 5C) were increased at 1-month cryopreservation and CD4^−^CD8^−^ T cells (11.8% increase, *p* = 0.0222) (Fig. 5E) were increased at 6-month cryopreservation compared to fresh samples.

For NK cell subsets, no significant changes in population frequencies were observed between fresh and 1- or 6-month cryopreserved samples for CD16^+^CD56^bright^ (Fig. 5F), CD16^+^CD56^dim^ (Fig. 5G) and CD16^−^CD56^bright^ (Fig. 5H) NK cells. Similarly, the population frequencies of total CD19^+^ B cells (Fig. 5I) or classical (Fig. 5J), intermediate (Fig. 5K) and non-classical (Fig. 5L) monocytes did not significantly change after 1- or 6-month cryopreservation.

### Cryopreservation of PBMCs for up to 6 months causes statistically significant changes in population frequencies of CD39^+^CD19^+^ B cells and CD39^+^ non-classical monocytes

**Fig. 6:**
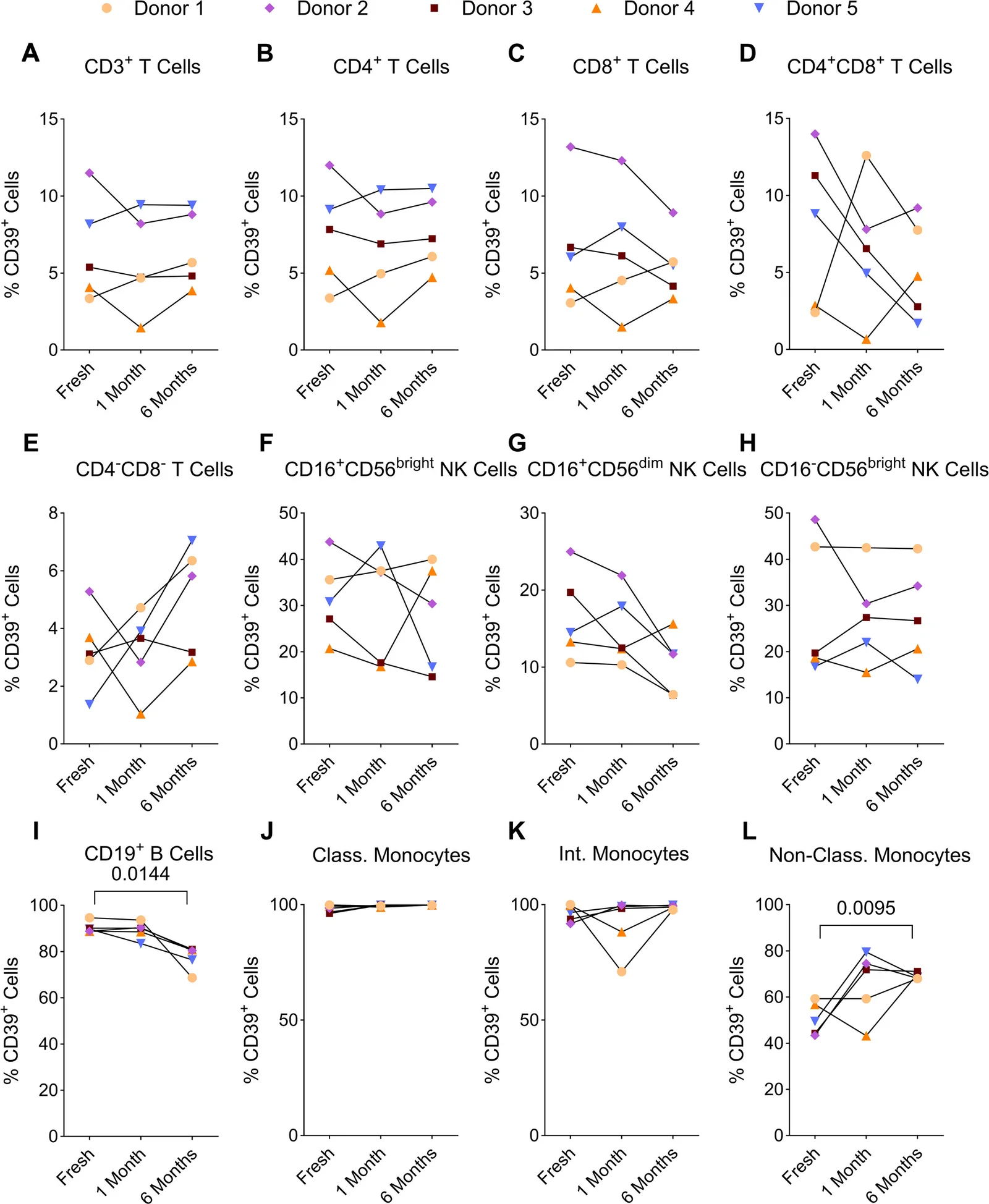
Cryopreservation of PBMCs for up to 6 months causes statistically significant changes in population frequencies of CD39^+^CD19^+^ B cells and CD39^+^ non-classical monocytes. PBMCs from five healthy donors were freshly isolated from whole blood using SepMate density gradient separation and analysed either immediately (fresh) or after cryopreservation for 1 or 6 months. Fresh or cryopreserved PBMCs were stained with Zombie NIR live-dead stain and fluorochrome-conjugated mAbs and analysed by flow cytometry. Values represent the percentage of CD39^+^ cells within each respective cell population. Data were tested for normality using a Shapiro–Wilk test. Normally distributed data were analysed using a (**A**-**H, K**-**L**) repeated-measures one-way ANOVA with Geisser-Greenhouse correction and a Dunnett’s multiple comparisons test. Non-normally distributed data were analysed using a (**I**, **J**) Friedman test, followed by a Dunn’s multiple comparisons test

To determine the impact of cryopreservation on CD39^+^ immune cells, the percentage of CD39^+^ cells within each immune cell subset studied above (Fig. 5) was determined by flow cytometry using a consistent gating strategy (Fig. 4E, not shown). CD39^+^ cells were present in varying amounts across all immune cell populations at all three timepoints. The frequency of CD39^+^ cells amongst each T cell subset was less than 15% (Fig. 6A-6D), except for CD4^−^CD8^−^ T cells, which was less than 8% (Fig. 6E). The frequency of CD39^+^ cells amongst CD16^+^CD56^bright^ (Fig. 6F) and CD16^−^CD56^bright^ (Fig. 6H) NK cells were each less than 50%, while the frequency of CD39^+^ cells amongst CD16^+^CD56^dim^ NK cells was less than 30% (Fig. 6G). The frequency of CD39^+^ cells amongst total CD19^+^ B cells was less than 95% (Fig. 6I). The frequency of CD39^+^ cells amongst classical monocytes (Fig. 6J) and intermediate monocytes (Fig. 6K) was near 100%, but less than 80% for non-classical monocytes (Fig. 6L).

The percentage of T cell, NK cell and monocyte subsets and B cells following cryopreservation for 1 or 6 months was then compared to fresh samples. No significant changes in the frequency of CD39^+^ cells in each T cell subset were observed between fresh and cryopreserved samples (Fig. 6A-6E). Likewise, no significant changes in the frequency of CD39^+^ cells in each NK cell subset were seen between fresh and cryopreserved samples of NK cell subsets (Fig. 6F-H). In contrast, a statistically significant decrease was observed in the frequency of CD39^+^ cells in CD19^+^ B cells at 6-month cryopreservation (14.3% decrease, *p* = 0.0144) (Fig. 6I). No significant changes were seen in the frequency of CD39^+^ cells in classical (Fig. 6J) or intermediate (Fig. 6K) monocytes between fresh and cryopreserved samples. Conversely, a statistically significant increase was observed in the frequency of CD39^+^ cells in non-classical monocytes at 6-month cryopreservation (36.6% increase, *p* = 0.0095) (Fig. 6L).

## Discussion

This study aimed to assess the impact of cryopreservation on the population frequencies of immune cell subsets including CD39^+^ cells in human PBMC samples. The findings of this study indicated that cryopreservation induced mostly minor, yet statistically significant changes in some population frequencies including CD39^+^ cells in human PBMC samples*.* Specifically, cryopreservation for up to 6 months had a minimal impact on the population frequency of human PBMCs, although minor but statistically significant changes were observed in CD4^+^, CD8^+^ and CD4^−^CD8^−^ T cells. Further, cryopreservation for up to 6 months had a minimal impact on the population frequencies of CD39^+^ cells, except for B cells and non-classical monocytes. Moreover, using transfected HEK293 cells, the study validated the use of the anti-human CD39 mAb (clone TU66) against human CD39 but showed that the anti-human P2X7 mAb (clone 1058613) was unable to selectively bind human P2X7.

The findings of this study showed that the population frequencies of T cell subsets changed slightly after cryopreservation, except for CD4^+^CD8^+^ and total CD3^+^ T cells, which remained unchanged. This agrees with a prior study reporting that T cells are the PBMC subset most affected by cryopreservation [36]. This former study also reported that CD4^+^ T cells were particularly susceptible to cryopreservation due to an increased production of reactive oxygen species, leading to cell death. The observed decrease in the frequency of CD4^+^ T cells may in turn have contributed to the increase in frequency of CD8^+^ T cells, as both subsets are determined as a percentage of total CD3^+^ T cells. The population frequency of CD4^−^CD8^−^ T cells was slightly increased after 6-month cryopreservation. This finding reflects the capacity of CD4^−^CD8^−^ T cells to maintain viability and function after cryopreservation, as previously shown [37]. No significant changes were found in the population frequencies of each NK cell subset after cryopreservation for up to 6 months, agreeing with previous studies [38, 39]. Similarly, no significant changes were found in the population frequencies of B cells or monocyte subsets. This agrees with prior studies that reported stability in population frequencies of cryopreserved B cells [40] and monocytes [41].

Before assessing the impact of cryopreservation of CD39^+^ immune subsets, this study validated the use of the anti-human CD39 mAb (clone TU66) by flow cytometry. While this mAb has been extensively used to study CD39 on immune cells [28–30], to the best of our knowledge, the binding of this mAb to CD39 has not been validated independently of the companies which produce this mAb. Here, the anti-CD39 mAb bound to human CD39 but not mock-transfected HEK293, indicating specific binding to human CD39 in these cells. Therefore, this mAb was subsequently used to assess the frequency of CD39^+^ immune cell subsets and the impact of cryopreservation on these cells.

Findings showed that CD39 was present on the surface of all studied immune cell populations, although in varying amounts. Low frequencies of CD39^+^ cells were observed amongst T cell subsets (all less than 15%, with CD4^−^CD8^−^ T cells less than 8%). This is consistent with a prior study describing comparable frequencies of CD39^+^CD4^+^ and CD39^+^CD8^+^ T cells [42]. Data on CD39^+^CD4^+^CD8^+^ and CD39^+^CD4^−^CD8^−^ T cells remain limited. However, as CD4^+^ and CD8^+^ T cells collectively represent ~ 90% of total T cells and previously displayed similar CD39^+^ frequencies to the current study [42, 43], the comparable low frequencies observed in the CD4^+^CD8^+^ and CD4^−^CD8^−^ T cell subsets are consistent with these prior findings. Another prior study reported similar low CD39^+^ frequencies for CD4^−^CD8^−^ T cells [44], further supporting current observations. Moderate frequencies of CD39^+^ cells were observed in CD16^+^CD56^bright^ and CD16^−^CD56^bright^ NK cells (both less than 50%), and in CD16^+^CD56^dim^ NK cells (less than 30%). This is consistent with prior studies reporting relatively higher frequencies of CD39^+^ cells in CD56^bright^ NK cells compared with CD56^dim^ NK cells [45, 46]. The frequency of CD39^+^CD19^+^ B cells (~ 90%) agreed with prior studies [42, 47]. High frequencies of CD39^+^ classical monocytes (~ 99%) and CD39^+^ intermediate monocytes (~ 96%), but a lower frequency of non-classical monocytes were observed. Given that classical monocytes represent approximately 80% of total monocytes, these CD39 findings are similar to those previously reported for total monocytes [42, 48, 49]. Another previous study also reported a lower frequency of CD39^+^ non-classical monocytes compared with classical monocytes [50], which again is consistent with the current study.

Cryopreservation for up to 6 months had no significant impact on CD39^+^ cells amongst all immune cell subsets, with two exceptions. First, the frequency of CD39^+^ B cells was decreased at 6-month cryopreservation. This observation aligns with a previous study in our group showing a decrease in the frequency of CD39^+^CD73^−^ B cells after 3-month cryopreservation [22]. This effect on CD39^+^ B cells is not common to all cell-surface molecules, as others have reported a minimal impact of cryopreservation on various B cell molecules [41] and the frequency of CD19^+^ B cells was not significantly altered in the current study. Second, the frequency of CD39^+^ non-classical monocytes was increased at 6-month cryopreservation. Again, this effect is not common to all cell-surface molecules, as others have reported a minimal impact of cryopreservation on other monocyte molecules [51], and the frequency of each monocyte subset was not significantly altered in the present study. To the best of our knowledge, this study is the first to report the impact of cryopreservation on the population frequency of CD39^+^ cells in CD4^+^CD8^+^ and CD4^−^CD8^−^ T cells, and NK cell and monocyte subsets. In contrast to the two exceptions above, cryopreservation had minimal impacts on CD39^+^ T cell and NK cell subsets, as well as CD39^+^ classical and intermediate monocytes. This supports the notion that cryopreservation is a reliable way of preserving cells for the study of CD39^+^ immune subsets, but changes should be considered on some subsets, especially B cells.

In contrast to the binding of an anti-CD39 mAb (clone TU66) to human CD39, this study was unable to verify the binding of a recent commercially available anti-P2X7 mAb (clone 1058613) to human P2X7. This mAb bound similarly to both non-transfected, P2X7-null HEK293 cells and HEK-P2X7 cells. Conversely, under the same staining conditions, the anti-P2X7 mAb (clone L4) bound to HEK-P2X7 cells but not non-transfected HEK293 cells, consistent with prior findings [34, 35]. Collectively, this suggests that the anti-P2X7 mAb (clone 1058613) does not selectively detect human P2X7 under the staining conditions employed in this study, and as such is unable to discriminate between P2X7^+^ and P2X7^−^ cells. This cautions against the use of this mAb to study P2X7 without further validation and highlights the importance of validating other new mAbs targeting P2X7 and other purinergic molecules before their use in flow cytometric and other studies.

This study indicates several future research directions. PBMCs that are frozen and immediately thawed and assessed by flow cytometry may help determine if any changes observed in cells are related to the freeze–thaw process rather than cryopreservation per se. The impact of cryopreservation on PBMCs from blood samples obtained from clinics, biobanks or blood banks should be examined to reflect the various sources of samples used in biomarker and other studies. Furthermore, blood samples from a larger number of subjects and a wider donor pool should be considered to account for potential differences in sex, ethnicity, diet or environment. Moreover, given that many studies use PBMCs cryopreserved for several years, much longer timeframes of cryopreservation should be considered. Finally, reactive oxygen species production in CD4^+^ T cells and other cell subsets following cryopreservation should be explored to confirm a role for these molecules in any changes because of cryopreservation.

To conclude, this study shows that cryopreservation can have mostly minor, yet statistically significant changes in the population frequencies of some immune cell subsets including CD39^+^ cells in PBMCs. These changes should be considered when interpreting data from immune cell analyses using cryopreserved PBMC samples, especially in studies focusing on small differences or on CD39^+^ B cells. Furthermore, this study validated the use of the anti-CD39 mAb (clone TU66) for the flow cytometric detection of human CD39, but not the anti-P2X7 mAb (clone 1058613) for the flow cytometric detection of human P2X7.

## Supplementary Information

Below is the link to the electronic supplementary material.

Supplementary file1 (DOCX 548 KB)

## Data Availability

All article data supporting the findings of this study including raw data is available upon reasonable request to the corresponding author.

## Abbreviations

ADP: Adenosine 5’-diphosphate
AMP: Adenosine 5’-monophosphate
ATP: Adenosine 5’-triphosphate
APC: Allophycocyanin
DMEM/F12: Dulbecco’s Modified Eagle Medium/Nutrient Mixture F12
DMSO: Dimethyl sulfoxide
EDTA: Ethylenediaminetetraacetic acid
FBS: Foetal bovine serum
FMO: Fluorescent minus one
HEK-P2X7: HEK293 cells stably transfected with human P2X7
HEK: Human embryonic kidney
mAb: Monoclonal antibody
MFI: Median fluorescent intensity
NK: Natural killer
PBS: Dulbecco’s phosphate-buffered saline
PBMCs: Peripheral blood mononuclear cells
RPMI: Roswell Park Memorial Institute
RT: Room temperature
