# Supplementary material for: Impact of cryopreservation on immune cell subset frequencies in human peripheral blood mononuclear cells following assessment of monoclonal antibodies to CD39 and P2X7
Source: Purinergic Signal. 2026 Sep 7;22(5):68. doi: 10.1007/s11302-026-10180-4 (PMC13550357; doi:10.1007/s11302-026-10180-4)
Supplement: Supplementary file 1 [file 11302_2026_10180_MOESM1_ESM.docx]

**Fig S1** Representative gating strategy to identify HEK293 cells. Live cells were gated by using Hoechst 33342 nucleic acid stain and forward-scatter area (FSC-A). Single cells were gated by FSC-A and height (FSC-H). FSC-A and side-scatter area (SSC-A) were used to gate HEK293 cells

**Fig S2** Titration of the anti-human P2X7 mAb (clone 1058613) with mixed HEK293 and HEK-P2X7 cells showed maximal binding at 1 to 2 µg per test. Non-transfected HEK293 and HEK-P2X7 cells were mixed 1:1 and incubated in the absence (FMO) or presence of 0.02 µg, 0.05 µg, 0.1 µg, 0.25 µg, 0.5 µg, 1 µg or 2 µg of the Alexa Fluor 647-conjugated anti-human P2X7 mAb (clone 1058613) and Hoechst 33342 nucleic acid stain and analysed by flow cytometry. (**A**) The marker was set to ~2% positive cells for each FMO group, with the difference between mAb binding and the corresponding FMO used to determine the percentage of P2X7^+^ cells. (**B**) The difference in MFI between the anti-P2X7 mAb and the FMO was used to determine anti-P2X7 mAb binding to human P2X7. (**A**, **B**) Results are from one experiment
